# A Textbook of Chemistry for Students of Medicine, Pharmacy and Dentistry

**Published:** 1903-12

**Authors:** 


A TEXTBOOK OF CHEMISTRY FOR STUDENTS OF MEDICINE, PHARMACY AND DENTISTRY. By EDWARD CURTIS HILL, M. S., M. D., Professor of Chemistry and Metallurgy in the Colorado College of Dental Surgery; Professor of Chemistry and Toxicology in Denver and Gross College of Medicine, University of Denver. Octavo, pp. 535. With 78 illustrations, including 9 full-page half-tone colored plates. Philadelphia: F. A. Davis Company. 1903. (Cloth, $3.00.)

Upon looking over the table of contents one is struck by the wide range of information which the author attempts to cover in the space of 510 pages. A careful reading of this book shows, however, that condensation has been carried to the extreme, and that in many instances the subjects have been merely mentioned, while in others the knowledge imparted is very meager.

The first section treats of medical physics, and to our mind is the freest from errors of any in the book. To be in strict accord with the terminology used in the balance of the work the term "medic" should have been used in place of "medical."

The too early use of symbols and formulæ, almost before the student has become acquainted with their meaning or significance, tends to confuse the student and render the subject more difficult. If less attention had been given to an effort to cover a wide field, and more to clearness and accuracy of statement, much might have been accomplished.

On page 93 the author gives the melting point of silver as 1040°, and on page 96 as 954°. Speaking of alum, on page 164, the author says: "Soda alum is much used as the acid element in cheap baking powders. This practice is reprehensible, since part of the salt changes in the stomach to phosphate and hydrate of al., both of which are soluble in the gastric juice, and the like." From the statement as made the student is left to conjecture as to the source of the phosphoric acid which would produce this phosphate.

On page 182 is said: "IDENTIFICATION OF CYANATES.—On moistening they yield the corresponding bicarbonate." Comment is unnecessary upon a statement so much at variance with facts. On page 193 the writer says that benzin, benzolin, or petroleum, ether remains semiliquid at 175°, while 12 lines above this statement, the boiling point of benzin is given as 50°-60°. Page 204, "aristol is an odorless substitute for C $H_3$." Comment again is unnecessary.

Speaking of hydrocyanic acid, on page 354, the author says: "Chlorin-water and calcium chlorid are mentioned as antidotes." We know of no author who mentions calcium chlorid as an antidote for prussic acid poisoning. Chloride of lime or calcium hypochorite is mentioned and has been used with good results. Under the heading of "Poisonous Bites and Stings," the writer again makes the same error.

References to careless and inaccurate statements might be multiplied, but we believe a sufficient number have been given to indicate the general character of the work before us.

On the whole, we do not feel justified in recommending this work for general use. From the publishers' standpoint the book is well printed and the plates are good. J. A. M.

